# Treatment of HIV-Infected Individuals with the Histone Deacetylase Inhibitor Panobinostat Results in Increased Numbers of Regulatory T Cells and Limits *Ex Vivo* Lipopolysaccharide-Induced Inflammatory Responses

**DOI:** 10.1128/mSphere.00616-17

**Published:** 2018-02-14

**Authors:** Christel Rothe Brinkmann, Jesper Falkesgaard Højen, Thomas Aagaard Rasmussen, Anne Sofie Kjær, Rikke Olesen, Paul W. Denton, Lars Østergaard, Zhengyu Ouyang, Mathias Lichterfeld, Xu Yu, Ole Schmeltz Søgaard, Charles Dinarello, Martin Tolstrup

**Affiliations:** a Department of Infectious Diseases, Aarhus University Hospital, Aarhus, Denmark; b Department of Clinical Medicine, Aarhus University, Aarhus, Denmark; c Ragon Institute of MGH, MIT and Harvard, Cambridge, Massachusetts, USA; d Department of Medicine, University of Colorado Denver, Aurora, Colorado, USA; e Department of Internal Medicine, Radboud University Medical Centre, Nijmegen, The Netherlands; Icahn School of Medicine at Mount Sinai

**Keywords:** T-cell immunity, clinical trials, gene expression, histone deacetylase inhibitors, human immunodeficiency virus, inflammation

## Abstract

Histone deacetylase inhibitors (HDACi) modulate the transcriptional activity of all cells, including innate and adaptive immune cells. Therefore, we aimed to evaluate immunological effects of treatment with the HDACi panobinostat in HIV-infected patients during a clinical phase IIa latency reversal trial. Using flow cytometry, we investigated changes in T cell activation (CD69, CD38, HLA-DR) and the expression of CD39 and CTLA4 on regulatory T cells (Tregs). Whole-blood stimulations were performed and cytokine responses measured using Luminex. Gene expression in purified peripheral blood mononuclear cells (PBMCs) was evaluated using an Affymetrix HTA 2.0 gene chip. We found that proportions of CD4^+^ and CD8^+^ T cells expressing CD69 increased 24 h after initial panobinostat administration (*P* < 0.01), followed by an increase in the proportions of CD38^+^ HLA-DR^+^-coexpressing CD4^+^ T cells on day 4 (*P* = 0.02). Concurrently, proportions of Tregs increased by 40% (*P* = 0.003). Treg CTLA4 median fluorescent intensity (MFI) increased by 25% (*P* = 0.007), and CD39 MFI on CD39^+^ Treg increased by 12% (*P* = 0.02). Lipopolysaccharide (LPS)-induced inflammatory responses (interleukin-1β [IL-1β], IL-6, IL-12p40, and tumor necrosis factor alpha [TNF-α]) in whole blood were significantly downregulated 4 days after initial dosing. Lastly, panobinostat induced significant changes in the overall gene expression pattern (fold change, >1.5; false-discovery-rate [FDR]-corrected *P*, <0.05). Importantly, measures of immune function returned to baseline after panobinostat treatment and follow-up revealed no sustained effect on overall gene expression.

**IMPORTANCE** The effect of treatment with histone deacetylase inhibitors on the immune system in HIV-infected individuals is not clear. Analysis of results from a clinical trial in which 15 HIV-infected individuals received 12 doses of panobinostat identified a significant impact on both T cell activation status and regulatory T cell suppressive marker expression and a reduced level of monocytic responsiveness to inflammatory stimuli. These changes were substantiated by global gene expression analysis. Collectively, the results suggest that panobinostat has multiple effects on innate and adaptive immune responses. Importantly, all the effects were transient, and further panobinostat treatment did not cause persistent long-term changes in gene expression patterns in HIV-infected individuals.

## Abstract (precis)

The effect of treatment with histone deacetylase inhibitors on the immune system in HIV-infected individuals is not clear. Analysis of results from a clinical trial in which 15 HIV-infected individuals received 12 doses of panobinostat identified a significant impact on both T cell activation status and regulatory T cell suppressive marker expression and a reduced level of monocytic responsiveness to inflammatory stimuli. These changes were substantiated by global gene expression analysis. Collectively, the results suggest that panobinostat has multiple effects on innate and adaptive immune responses. Importantly, all the effects were transient, and further panobinostat treatment did not cause persistent long-term changes in gene expression patterns in HIV-infected individuals.

## INTRODUCTION

Histone deacetylase inhibitors (HDACi) are known for their ability to modulate the epigenetic regulation of multiple genes, which in the field of HIV eradication has been exploited to reactivate latent HIV (1). We recently conducted a clinical trial in HIV-1-infected patients on combination antiretroviral therapy (cART) to investigate the ability of the HDACi panobinostat to disrupt HIV-1 latency (2). Here, a successful reactivation of viral transcription by the use of panobinostat has established that this HDACi is a potential future latency reversal agent (LRA) suitable for use in a curative strategy. However, a comprehensive analysis of the impact of panobinostat on the immune system is a necessity for fully evaluating its potential in future clinical trials.

HDACi were originally developed for the treatment of hematologic malignancies by virtue of their capacity to cause growth arrest, differentiation, and apoptosis of tumor cells (3). The same is true for panobinostat, being a highly potent oral pan-HDACi recently approved for the treatment of multiple myeloma (4). HDACi reversibly inhibit the activity of histone deacetylases (HDACs) and thereby modify gene expression and inflammatory responses (5, 6). While the immunoregulatory properties of HDACi have considerable therapeutic potential to target pathological inflammation (7), the toxicities associated with the dosages used for cancer treatment have impeded the development of HDACi as immunomodulatory drugs in other diseases. However, it should be noted that the immunomodulatory effects of HDACi are elicited at much lower concentrations than those required for direct antitumor effects, expanding the range of their beneficial uses (8–11).

HDACi affect pathological tissue differently from normal tissue; e.g., malignant cells are more sensitive to HDAC inhibition than nonmalignant cells, and antineoplasm effects dominate over immunomodulatory effects when both cell types are present *in vivo* (12). The latter observation might explain why contradictory results arise from *in vitro* models, animal models, and human studies. In fact, the overall effect of HDACi treatment on host immune pathways appears to vary with both the applied model system and the indication for which HDACi is administered. Therefore, interpretation of preclinical data requires careful consideration and necessitates confirmation of findings in clinical trials. However, the clear majority of clinical HDACi trials have been conducted in the oncology field, and those studies have offered little insight into immunological effects of HDACi treatment in patients without malignancy. Hence, a deeper understanding of the immunomodulatory effects of HDACi in nononcology patients is much needed to guide the potential development of HDACi for the treatment of other disease indications such as their use in anti-inflammatory and curative HIV strategies.

Our recent clinical trial provided a unique opportunity to comprehensively evaluate the diverse immunomodulatory effects of panobinostat *in vivo* in the context of chronic HIV infection. We previously described the immunological impact of panobinostat on interferon-stimulated genes, HIV-specific cytokine production, and biomarkers of cardiovascular risk (13, 14). Thus, here we expand this immune cell profiling by reporting on T lymphocyte characterizations, innate immune responses, and extensive gene expression profiling in peripheral blood mononuclear cells (PBMCs). Providing this additional view into the immune system thereby offers invaluable information about the impact of panobinostats on HIV-1-infected patients to guide its future use as a component of HIV eradication efforts.

## RESULTS

### T cell activation.

**FIG 1:**
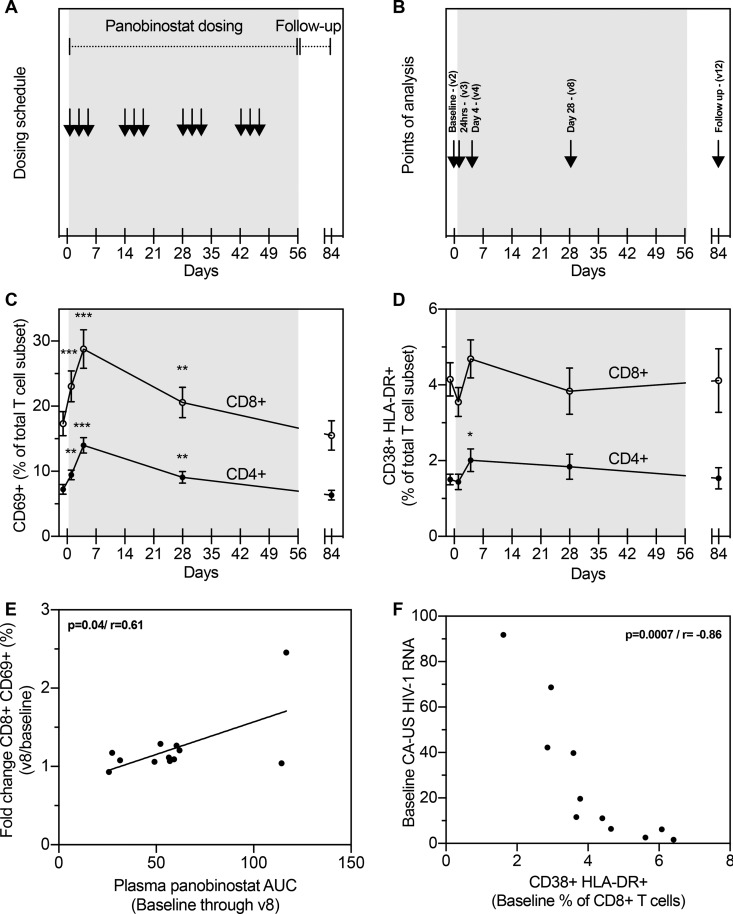
Impact of panobinostat on T cell activation and its possible relation to measurement of viral replication. (A) Study design depicting 20 mg panobinostat dosing three times per week every other week for a total of 8 weeks. (B) Time points of analysis outlined: baseline; 24 h after the first dosing; day 4 (8 h postdosing); day 28 (8 h postdosing); at follow-up on day 84, 4 weeks after last dosing. (C) Percentages of CD4^+^ and CD8^+^ T lymphocytes expressing the early activation marker CD69 during the study. (D) Percentages of CD4^+^ and CD8^+^ T lymphocytes expressing both late activation marker HLA-DR and activation marker CD38 during the study. (E) Correlation between CD8^+^ CD69^+^ fold change (visit8/baseline) and AUC for plasma panobinostat concentrations from baseline through v8. (F) Correlation between levels of CA-US HIV-1 RNA and CD38^+^ HLA-DR^+^ T cells. Changes in T cell expression patterns compared to baseline were evaluated by a paired *t* test. Correlations were evaluated using Pearson's correlation. Means and standard errors of the means (SEM) of results are depicted. *, *P* < 0.05; **, *P* < 0.01; ***, *P* < 0.001.

To determine the impact of repeated panobinostat dosing on overall T cell activation, we evaluated the expression of the early T cell activation marker CD69 throughout the study (Fig. 1A and B). At 24 h following panobinostat administration, we recorded an increase in the proportions of both CD4^+^ T cells (*P* = 0.002) and CD8^+^ T cells (*P* = 0.0005) expressing CD69 (Fig. 1C). CD69 expression was also elevated on day 28 (at the start of third treatment cycle) but returned to baseline levels for both CD4^+^ and CD8^+^ T cells 4 weeks after completion of panobinostat treatment (on-panobinostat). A similar pattern of increase, albeit occurring less promptly and in a less pronounced manner, was observed for CD38^+^ HLA-DR^+^ doubly positive T cells. We observed no change at 24 h after initiation of panobinostat. However, the proportion of T cells positive for CD38 and HLA-DR was modestly increased for CD4^+^ (but not CD8^+^) T cells on day 4 post-panobinostat initiation (*P* = 0.02) (Fig. 1D). Of note, no subset specific evaluation was done.

To assess the impact of plasma panobinostat concentrations (2) and their relation to T cell activation, we correlated levels of fold change in activation (baseline to day 4 and day 28) to area under the curve (AUC) values for plasma panobinostat in the same time frame, as a measure of total drug exposure impact. We observed a significant correlation of levels of fold change in CD69 expression between CD4^+^ (not shown) and CD8^+^ T cells (Fig. 1E) on day 28, whereas no significant correlation was observed for CD38^+^ HLA-DR^+^ T cells.

To investigate a possible relationship between T cell activation and viral transcription, we next correlated expression of activation markers to the previous investigated levels of cell-associated unspliced HIV-1 RNA (CA-US HIV-1 RNA) found in CD4^+^ T cells as a biomarker of viral transcriptional levels (previously published [2]). We found no significant correlations between CD4^+^ T cell activation and the level of transcription (data not shown). However, examining CD8^+^ T cell activation, we observed a significant inverse correlation (*P* < 0.0001) between the levels of transcription and of CD38^+^ HLA-DR^+^ T cells at baseline (Fig. 1F).

### The impact of panobinostat on Tregs.

**FIG 2:**
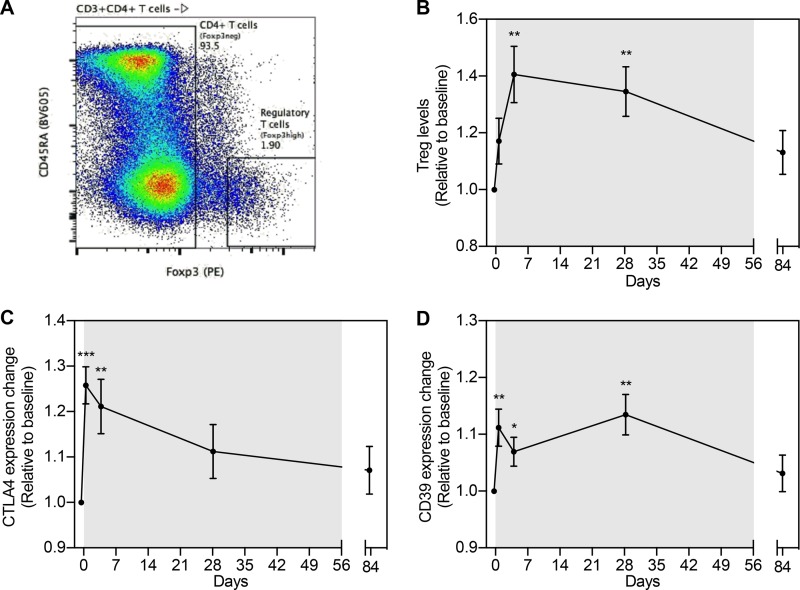
Panobinostat affects the levels of regulatory T cells and inhibitory receptor expression. (A) Gating definition of regulatory T cells (Treg). PE, phycoerythrin. (B) Relative changes in levels of Treg (CD4^+^ CD45RA^neg^ FoxP3^high^) during the course of panobinostat therapy. (C) Relative changes in intracellular expression of the suppressive marker CTLA4 in Treg. (D) Relative changes in expression of the suppressive marker CD39 on Treg. Changes in levels of expression (median fluorescence intensity [MFI]) compared to baseline were evaluated by a paired *t* test. Means and SEM are depicted. *, *P* < 0.05; **, *P* < 0.01; ***;*P* < 0.001.

We defined the regulatory T cell population as CD3^+^ CD4^+^ CD45RA^−^ and Foxp3^high^ (15) (Fig. 2A). The proportion of Tregs increased following panobinostat dosing, with a 40% increase on day 4 (*P* = 0.003). Levels of Tregs were also elevated on day 28 of panobinostat treatment (*P* = 0.004) but returned to baseline at follow-up (Fig. 2B). Furthermore, the median fluorescent intensities (MFI) of suppressive marker CTLA-4 (on total Tregs) and CD39 (on CD39^+^ Tregs) increased significantly in the first dosing week by 25% and 12%, respectively (Fig. 2C and D). The increase in CTLA4 expression shortly following panobinostat initiation was not maintained, as levels were comparable to baseline at day 28 on-panobinostat (Fig. 2C), in contrast to the level of Treg CD39 expression, which remained significantly elevated on day 28 on-panobinostat (*P* = 0.006) but returned to baseline at follow-up 4 weeks post-panobinostat initiation (Fig. 2D).

### Whole-blood responsiveness to LPS stimulation.

**FIG 3:**
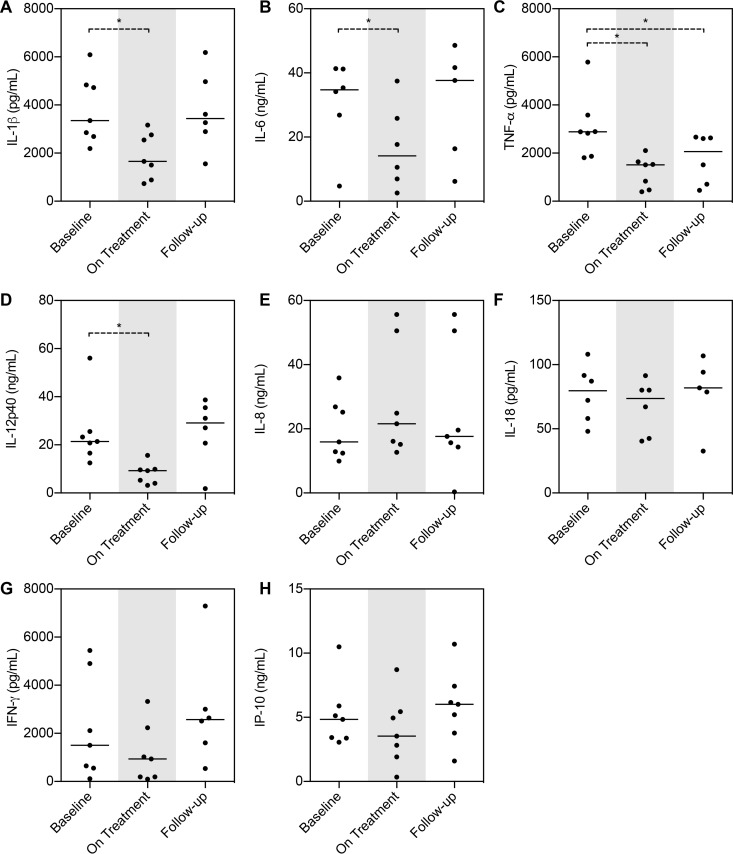
LPS induced cytokine production in whole blood inhibited by panobinostat treatment. LPS induced release of cytokines/chemokines from 24-h whole-blood stimulations at day 28 (On Treatment). Samples were available from 7 patients. The Wilcoxon matched-pair signed-rank test was used. Medians are depicted. *, *P* < 0.05; **, *P* < 0.01.

To determine functional changes in immune responsiveness, whole-blood stimulations were performed with lipopolysaccharide (LPS). The LPS responses, as assessed by levels of interleukin-1β (IL-1β), IL-6, tumor necrosis factor alpha (TNF-α), and IL-12p40 secretion, were all significantly downregulated by panobinostat (day 28) (Fig. 3A to D). In contrast, secretion of IL-8, IL-18, gamma interferon (IFN-γ), and IFN-γ-induced protein 10 (IP-10) in response to LPS stimulation was not affected by panobinostat treatment (Fig. 3E to H).

**FIG 4:**
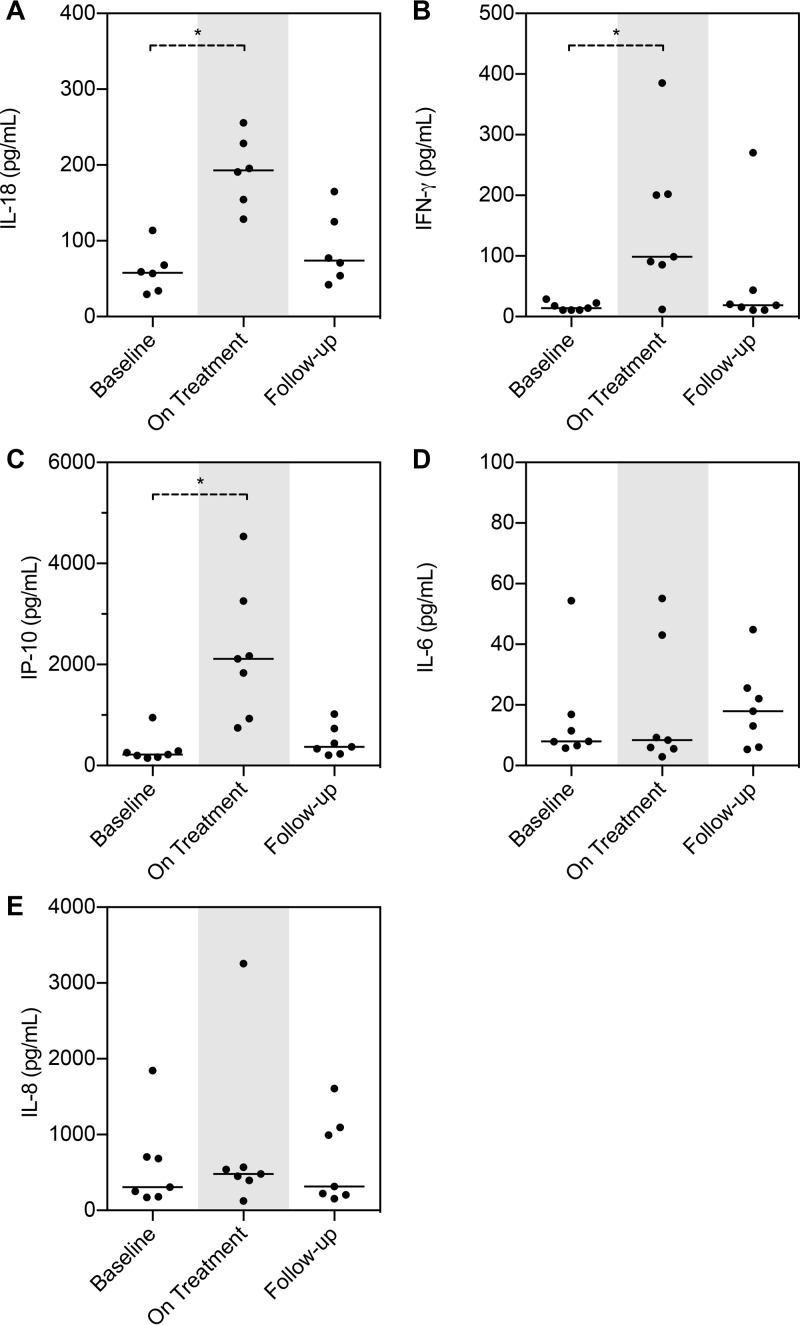
Spontaneous release of Th1 derived cytokines is influenced by panobinostat treatment. (A-E) cytokine/chemokine production from 24 h whole blood without LPS stimulation. TNF-α, IL-1β, and IFN-γ were all undetectable (data not shown). On Treatment, day 28. Samples were available from 7 patients. The Wilcoxon matched-pair signed-rank test was used. Medians are depicted. *, *P* < 0.05.

Analysis of control samples, i.e., whole blood not exposed to LPS but left untouched overnight, revealed an interesting pattern. Treatment with panobinostat primed a Th1 response with increased secretion of IL-18, IFN-γ, and IP-10 compared to baseline (*P* < 0.05; Fig. 4A to C). However, this priming did not represent a broad inflammatory activation response pattern, as IL-6 and IL-8 levels remained unchanged (Fig. 4D and E). TNF-α, IL-1β, and IL-12p40 were undetectable under all unstimulated conditions (data not shown).

### Change in epigenetic regulation of gene expression.

**FIG 5:**
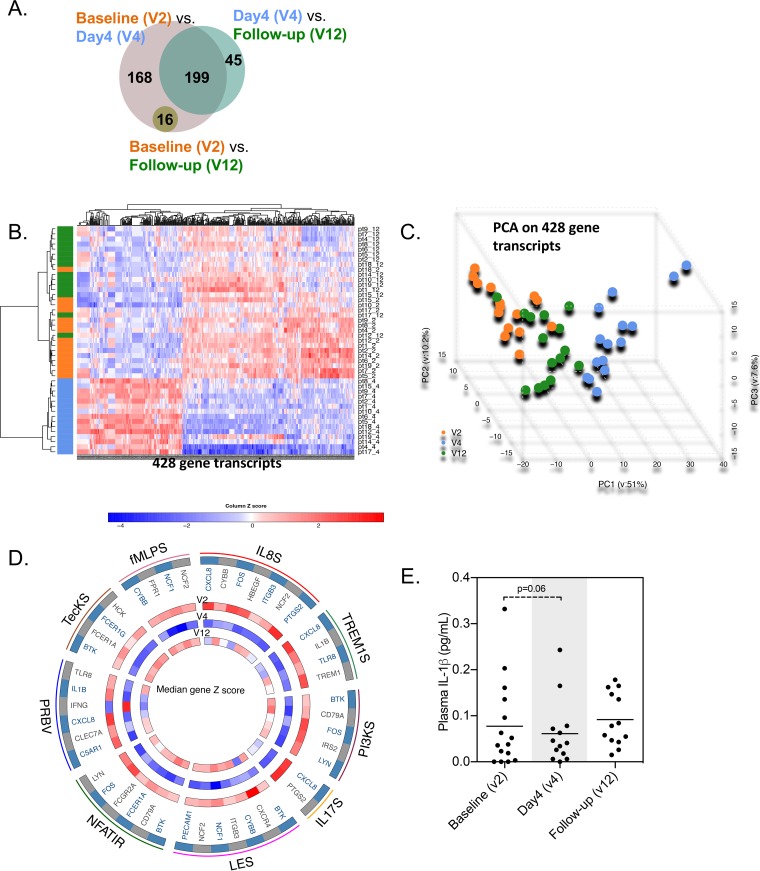
Panobinostat induces reversible perturbations in PBMC gene expression patterns. (A) Venn diagram summarizing the number of differentially expressed genes (DEG) in comparisons between the indicated study visits. V2, baseline; V4, day 4 of panobinostat treatment; V12, follow-up. (B and C) Heat map (B) and principal-component analysis (C) of 428 transcripts differentially expressed between V2, V4, and V12. Data points from V2, V4, and V12 are indicated in orange, blue, and green, respectively. (D) R-Circos plot summarizing functional pathways enriched for DEGs. Outer circles reflect selected functional pathways and corresponding DEGs (PRBV, role of pattern recognition receptors; NFATIR, NFAT-dependent immune regulation; LES, leukocyte extravasation signaling; IL17S, IL-17 signaling; PI3KS, PI3K signaling; TREM1S, TREM1 signaling; IL8S, IL-8 signaling; fMLPS, fMLP signaling; TecKS, tec kinase signaling). Inner circles indicate levels of gene expression intensity (median gene *z* scores) of the respective transcripts at the indicated study visits. (E) Plasma IL-1β levels (in picograms per milliliter). A paired *t* test was performed, and the means of the results are depicted.

We identified genes that were differentially expressed during treatment with panobinostat by considering three pairwise comparisons among three visits: baseline (visit 2 [V2]), day 4 on-panobinostat (V4), and follow-up 4 weeks post-panobinostat initiation (V12). We identified 383 gene transcripts that were differentially expressed between baseline and day 4, 244 transcripts that were differentially expressed between day 4 and post-panobinostat initiation follow-up, and only 16 transcripts that were differentially expressed between baseline and follow-up 4 weeks post-panobinostat initiation (Fig. 5A). Of note, no changes in gene expression were detected at an additional long-term follow-up visit 24 weeks post-panobinostat dosing (V13) compared to baseline (data not shown). Tight clustering of affected genes at day 4 on panobinostat is visualized in the heat map in Fig. 5B and in the principal-component analysis (PCA) depicted in Fig. 5C. Overall, these data show that panobinostat induced significant perturbations in gene expression patterns that were fully reversible after panobinostat treatment discontinuation. Notably, when we performed a functional annotation of genes differentially expressed during panobinostat dosing, we identified pathways related to a diverse spectrum of biological functions, including gene clusters involved in the regulation of pattern recognition pathways (PRBV [role of pattern recognition receptors]), IL-8- and IL-17-mediated signal transduction mechanisms (IL8S and IL17S), leukocyte extravasation signaling (LES), and the TREM1- and the phosphatidylinositol 3-kinase (PI3K) signaling cascades (Fig. 5D). Since caspase-1-mediated pyroptosis has been suggested to be an important contributor to CD4^+^ T cell depletion (16), it was an interesting finding that IL-1β expression was significantly decreased on-panobinostat. Investigating plasma concentrations of IL-1β using a high-sensitivity assay, we observed a tendency toward less production at day 4 on-panobinostat (*P* = 0.06) (Fig. 5E).

### Correlations between gene expression changes and CA-US HIV-1 RNA.

**FIG 6:**
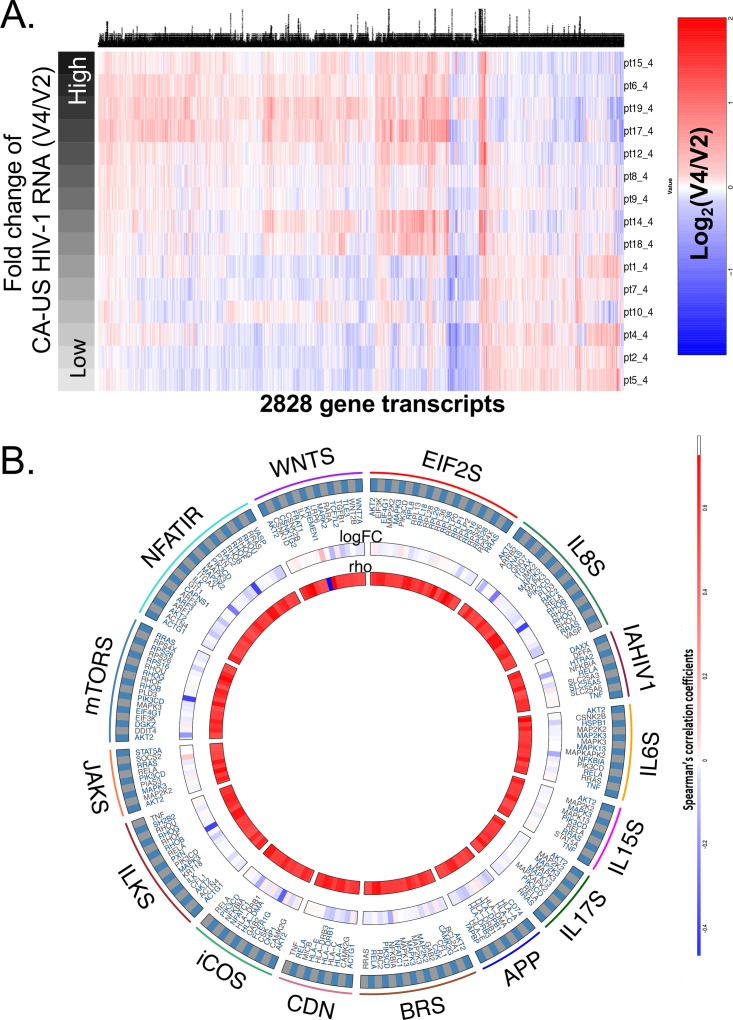
Gene expression changes associated with induction of cell-associated US HIV-1 RNA transcription during treatment with panobinostat. (A) Linear regression of fold changes between day 4 and baseline (V4 to V2) expressed in levels of gene expression intensity of host genes and corresponding changes in CA-US HIV-1 RNA. The heat map reflects all host transcripts correlated with CA-US HIV-1 RNA on a nominal *P* level of <0.05. Patients were ranked according to the intensity of CA-US HIV-1 RNA induction. (B) R Circos plot indicating gene sets enriched for host transcripts associated with CA-US HIV-1 RNA, as determined by linear regression calculated as described for panel A. Outer circles reflect the following gene set pathways: Wnts, Wnt signaling; NFATIR, NFAT-dependent immune regulation; mTORS, mTOR signaling; JAKS, JAK/STAT signaling; ILKS, ILK signaling; iCOS, iCOS/iCOL signaling; CDN, crosstalk between NK cells and dendritic cells; BRS, BCR signaling; APP, antigen presentation pathway; IL17S, IL-17 signaling; IL6S, interleukin-6 signaling; IAHIV1, induction of apoptosis by HIV; IL88S, interleukin-8 signaling; EIF2S, EIF2 signaling. Inner circles indicate fold changes (V4 to V2) for each individual transcript, and rho values (Spearman’s correlation coefficient) reflect the correlations with the corresponding CA-US HIV-1 RNA levels.

To further explore gene expression changes occurring during panobinostat treatment, we focused on transcriptional alterations between baseline and day 4 on-panobinostat. For this purpose, correlations between fold changes (binary logarithms [log_2_]) in levels of gene expression in PBMCs and corresponding fold changes in levels of CA-US HIV-1 RNA between baseline and day 4 were analyzed by Spearman’s correlation. A total of 2,828 gene transcripts were identified, with a nominal *P* value of <0.05. Transcripts strongly correlated with corresponding changes in CA-US HIV-1 RNA levels were functionally involved in cellular interactions mediated by IL-6, IL-8, IL-15, IL-18, and inducible costimulator/inducible costimulator ligand (iCOS/iCOSL), in antigen presentation and immune recognition pathways, and in beta-catenin and ILK signaling pathways (Fig. 6).

## DISCUSSION

In recent years, HDACi have been utilized in an increasing number of human studies targeting nonneoplastic diseases, including HIV-1 infection (5). However, comprehensive analyses of the impact of HDACi on the immune system are lacking, impeding efforts to design a more effective future method of use. In this study, we expanded our previous knowledge by exploring broad immunological changes induced during panobinostat dosing in HIV-1-infected individuals. Treatment with panobinostat resulted in a significant but transient increase in T cell activation, Treg proportions, and Treg expression of CTLA4 and CD39. Further, we observed lowered functional LPS responsiveness, with a countervailing Th1 phenotypic increase. The impact on innate signaling was rediscovered in the gene expression profiling, showing a marked transcriptional decrease on multiple innate signaling pathways. Importantly, all measures of immune function had returned to baseline levels 4 weeks after completion of panobinostat treatment, and follow-up examinations revealed no sustained effect on overall gene expression.

Several previous reports indicated that HDACi stimulation caused moderate T cell activation *in vitro* and *in vivo* (17–19), whereas other studies showed that HDACi inhibit the cytotoxic and proliferative capacity of activated or antigen-stimulated T cells (20, 21). Of note, previous data from our panobinostat clinical trial indicated no decrease in HIV-specific cytokine production as evaluated by intracellular cytokine staining (ICS) and enzyme-linked immunosorbent spot (ELISPOT) assays but instead a significant increase in the magnitude and breadth of the responsiveness of CD8^+^ T cells in the ELISPOT assay (13). In this study, panobinostat treatment resulted in a significant upregulation of surface activation markers on T lymphocytes, in line with results seen with other HDACi. CD69 is a transmembrane C-type lectin and one of the earliest proteins expressed after lymphocyte activation, with a key role in responses to inflammation and modulation of adaptive immune responses (22, 23). CD69 is tightly regulated at the transcriptional level, with nucleosome promoter positioning and histone H3 acetylation as important transcriptional determinants (24). Thus, the rapid induction of CD69 after dosing could be ascribed to a direct pharmacological effect of promoting histone acetylation or acetylation of proteins other than histones. Likewise, we simultaneously observed minor upregulations of CD38 and HLA-DR on CD4^+^ T cells. This increase could suggest a bona fide phenotypic activation of the lymphocyte compartment. In our observations, we also found that total panobinostat exposure over a longer time period (AUC, baseline through v8) correlated with the induction of CD69 expression on both CD4^+^ and CD8^+^ T cells. We were unable to decipher any direct impact of panobinostat or whether it is a secondary effect that subsequent initiates higher-level activation, for example, mediated by the higher level of HIV transcription as observed in this study.

The ability to activate the T cell compartment is an interesting observation in the context of inducing transcription of latent HIV-1. One of the key transcriptional regulators during T cell activation is the nuclear translocation of NF-κB, which is also an important determinant of efficient transcriptional initiation of the HIV-1 long terminal repeat (LTR) (25–27). We did not assess HDACi-induced NF-κB activation in this study; however, such an assessment would make an interesting observation in future clinical studies. The slightly enhanced activation of the CD4^+^ T cell compartment may contribute to the efficacy by which panobinostat induces expression of latent HIV-1 proviruses. Correlating the levels and changes in the levels of CA-US HIV RNA as a marker of transcription to the levels and changes in the levels of CD4^+^ T cell activation, we did not, however, find any significant correlations to support this hypothesis. This suggests that latency reversal can happen independently of T cell activation. Instead, a direct cell sorting of CD4^+^ T cells based on activation status with a subsequent analysis of transcriptional activity could be done in future clinical studies to address this specifically. Investigating the CD8^+^ T cells, we did, however, observe a highly statistically significant inverse correlation between the baseline transcriptional activity and the percentage of CD38^+^ HLA-DR^+^ T cells. CD8^+^ cytotoxic T cells have always been known to represent a sentinel cell type in the control of HIV (28). In an HIV-curative context, this seems to underline the importance of CD8^+^ T cells in controlling transcriptional activity and thus could potentially be used as a prestudy biomarker for individuals with a higher likelihood of being able to control virus.

We noted significant (up to 40%) expansion in the regulatory T cell population during panobinostat treatment. This observation is in line with previous reports describing vorinostat treatment in HIV-1-infected individuals (29) and in allogeneic hematopoietic stem cell transplantation (HSCT) recipients (30). Importantly, another study has shown that HDAC inhibition increases *Foxp3* gene expression, resulting in enhanced Treg suppressive function (31). In humans, inhibition of HDAC6 appears important for Treg induction (32). Both vorinostat and panobinostat have been shown to inhibit HDAC6 in the lower nanomolar range. In line with this, we observed increased expression of suppressive markers CD39 and CTLA-4 in the Treg population, and the latter has been shown to correlate tightly with Treg functional suppressive capacity (33). From an HIV-1 eradication perspective, the increase in Treg function during HDACi treatment could be counterproductive, given that Treg depletion has been shown to support disruption of viral latency (34), and may also contribute to inhibition of antiviral effector cells. Recently, a CTLA4^+^ PD-1^−^ T cell type that shares phenotypic markers with Tregs was shown to constitute a part of the HIV reservoir (35). The increase in levels of CTLA4 expression and Treg percentages that we observed could therefore be seen as potentially problematic in a context of homeostatic proliferation. The fact that we did not observe an increase in CD4^+^ total HIV DNA levels in the time frame of these peak incidences but instead observed a significant decrease (2) speaks against this concern. On the other hand, the clear induction of Treg may more reasonably be interpreted as a sign of a well-balanced unperturbed immune response counteracting the observed increased activation mediated by heightened T cell activation and Th1 priming.

HDACi have inhibitory effects on inflammatory cytokine production in cells of the innate immune system, e.g., monocytes, activated macrophages, and dendritic cells (10, 11, 36–39). A remarkable indication of this potential stems from a recent study in which a reduced dose of vorinostat (100 or 200 mg) added to the standard immunoprophylaxis for graft-versus-host disease (GVHD) following hematopoietic stem cell transplantation (HSCT) was associated with suppression of inflammatory cytokines, an increase in Treg, and an incidence of severe acute GVHD that was lower than expected (40). To characterize the immune responsive capacity during panobinostat treatment, we therefore performed whole-blood stimulations on freshly drawn blood before, during, and after dosing. In contrast to dissection of all cell types and responses, whole-blood stimulations provide a “true-to-nature” assay that includes all cell types in relevant concentrations, soluble receptors, and cytokines, essentially representing a complete peripheral immunological replicate. In the light of the impact of microbial translocations on chronic activation of the immune system in HIV-infected individuals, we chose to use stimulation with LPS as a classic indicator to establish whether panobinostat could attenuate this repeated microbial activation, benefiting the fitness of the immune system. Stimulation with LPS revealed a diminished inflammatory response indicated by decreased secretion of IL-1β, L-6, TNF-α, and IL12p40. This is consistent with our previous report showing an anti-inflammatory effect of panobinostat treatment as evidenced by significant decreases in plasma levels of C-reactive protein (CRP) and sCD40L (14). These decreased inflammatory responses, in conjunction with the analyses of the gene expression data showing a significant reduction in levels of genes associated with pattern recognition receptor responses (such as Toll-like receptor 4 [TLR4]), suggest a powerful modulatory effect of panobinostat on these innate immune pathways. Such modifications in the inflammatory responses following TLR4 stimulation have also been reported with givinostat (ITF2357) (10) and vorinostat (30). Other intriguing findings emerged from the whole-blood stimulations. In PBMCs of unstimulated whole blood, we found a significant upregulation of IFN-γ as well as IP-10 and IL-18. This is strongly indicative of a primed Th1 response and was corroborated by the finding that the IFN-γ gene was one of the single genes with the greatest fold change increase in the gene expression analysis. In that context, we previously reported ELISPOT assay results showing HIV-specific IFN-γ increases in both magnitude and breadth after panobinostat treatment, which further validates the corresponding findings. IFN-γ is important in induction of cellular immune effector functions (i.e., cytotoxic T cells and/or natural killer [NK] cells, both of which are believed to be key components in the pursuit of an effective HIV eradication strategy). That we have observed IFN-γ to be upregulated by panobinostat in multiple assays does represent a unique feature of this HDACi.

What is also evident from the data presented here is the profound impact of panobinostat on multiple innate and adaptive components of the immune system. We observed that global transcriptional signatures in PBMCs during panobinostat treatment showed highly distinct patterns, with changes in a broad spectrum of immune pathways, including pattern recognition pathways. While many of these transcriptional changes likely reflect unspecific effects related to HDACi-induced epigenetic variations in transcriptional control mechanisms, we also identified 2,828 transcripts with transcriptional alterations that closely correlated to corresponding changes in CA-US HIV-1 RNA levels. Of note, such transcripts were enriched for genes influencing cell-to-cell interaction and intercellular communication and were involved in innate immune recognition and antigen presentation pathways, iCOS/iCOSL-dependent cell signaling events, and intercellular communication mediated by IL-6, IL-8, IL-15, and IL-18. Together, these data invite the hypothesis that, in addition to cell-intrinsic direct pharmacological effects of HDACi on HIV-1 transcription, HDACi may also influence HIV-1 gene expression by indirect mechanisms that result from altered cellular interaction pathways and from changes in microenvironmental signals. Precisely how such altered cell-to-cell interactions may possibly contribute to viral reactivation during HDACi treatment represents an important area of investigation in future studies. In addition, the observed decreases in both IL-1β gene expression and protein plasma concentrations could suggest additional beneficial impacts of panobinostat. Given the hypothesis of pyroptosis mediating CD4^+^ T cell death, an inhibition of caspase-1 activity in CD4^+^ T cells would make the use of this HDACi highly attractive. One HDACi with this ability, givinostat (ITF2357), has already been reported to inhibit caspase-1 activity in stimulated PBMCs (41).

Despite addressing important aspects relevant for future HDACi utility, certain limitations should be noted. Primarily, due to the lack of a control group, we are unable to account for naturally occurring longitudinal variations in our measurements. However, the timing and rapidity of changes in both innate and adaptive compartments closely coinciding with the panobinostat administration are strongly suggestive of a causal relationship. In addition, as the gene expression analysis was made using total PBMCs, no immune compartment-specific impact can be inferred.

In conclusion, we found that treatment of HIV-1-infected individuals with the HDACi panobinostat significantly but transiently influenced T cell activation, increased proportions of Treg, and decreased functional mitogen responsiveness. Panobinostat also affected gene expression and impacted immune pathways. Alterations in immune functions normalized within 4 weeks after stopping panobinostat treatment and a long-term follow-up revealed no sustained effect on overall gene expression. Collectively, these results suggest that panobinostat causes neither persistent transcriptional changes nor persistent immunomodulatory changes in HIV patients.

## MATERIALS AND METHODS

### Study design.

We performed a single-arm, phase 1/2 trial at the Department of Infectious Diseases, Aarhus University Hospital, Aarhus, Denmark (ClinicalTrials registration no. NCT01680094). Briefly, 15 HIV-infected adults on suppressive cART (<50 copies per ml for at least 2 years) with a CD4 count above 500 cells per μl were enrolled (2). Study participants received oral panobinostat (20 mg) three times per week every other week for a total of 8 weeks while maintaining cART coverage (Fig. 1A). Blood sampling was done at multiple time points before, during, and after panobinostat administration. Samples used for this study were collected at baseline (day 0 [visit 2]), day 1 (24 h after the first dose [visit 3]), day 4 (8 h after third dose [visit 4]), day 28 (8 h after the seventh dose [visit 8]), and day 84 (4 weeks after the last dose [visit 12]) (Fig. 1B). Additional detailed information on inclusion and exclusion criteria has previously been published (2). The study was approved by the Danish Research Ethics Committee system and conducted in accordance with the principles of the Declaration of Helsinki. Each participant provided written informed consent before initiation of any study procedures.

### T cell activation and Treg.

Using flow cytometry, we evaluated both T cell activation status and suppressive marker expression on Treg throughout the study. For assessment of activation status, we investigated the activation markers CD69, HLA-DR, and CD38 (for Treg) and the suppressive capacity markers CD39 and CTLA4.

For analysis of T cell activation status, 4 × 10^5^ cryopreserved PBMCs were thawed and immediately stained with a viability dye (near-infrared [near-IR]; Life Technologies) for 30 min on ice. Afterward, nonspecific binding was blocked using 10% fetal bovine serum (FBS) and the cells were subsequently surfaced stained (30 min, 4°C) with antibodies to CD3 (SK7; BioLegend), CD4 (RPA-T4; BD), CD8 (SK1; BD), CD69 (fn50; BioLegend), CD38 (HB7; BD), and HLA-DR (G46-6; BD). Gates for activation marker positivity were determined using isotope control antibodies. For the Treg setup, we followed the same procedure, using 1 × 10^6^ cryopreserved PBMCs and antibodies to CD3 (SK7; BioLegend), CD4 (OKT4; BioLegend), CD45RA (HI100; BD), CD25 (M-A251; BD), and CD39 (A1; BioLegend). In addition, cells were fixed, permeabilized (transcription factor buffer set; BD), and subjected to intracellular staining (45 min, 4°C) with FoxP3 (236a/E7; BD) and CTLA4 (BNI3; BD). Treg were defined as CD4^+^ CD45RA^−^ FoxP3^high^ T cells. Foxp3^intermediate^-expressing CD4^+^ T cells were not included in the analyses, as they do not exhibit the same immunosuppressive properties (15).

Both setups were run on a FACSVerse flow cytometer (BD Biosciences) with the use of CompBeads (BD Biosciences) for compensation. All antibodies were titrated before use, and all analyses were performed in FlowJo (ThreeStar, NJ, USA).

### Cytokine production in whole-blood stimulations and plasma.

Whole-blood stimulations were performed to reveal any changes in the combined-response potential of cells in the peripheral blood. We used 250 µl of freshly harvested blood (heparin) transferred to two separate microcentrifuge tubes. Subsequently, the blood volumes in both tubes were diluted four times by adding 750 μl RPMI medium; one of the tubes included a final concentration of 100 ng/ml of lipopolysaccharide (LPS; Sigma). Both tubes were incubated overnight at 37°C in the incubator with the lid open to allow CO_2_ buffering. The following day, supernatants were gently harvested and immediately frozen at −80°C. We evaluated the production of IL-1β, IL-6, IL-8, IL-12p40, IP-10, IL-18, TNF-α, and IFN-γ on a Luminex 100 platform, using multiplex antibodies from Bio-Rad as recommended by the manufacturer. Whole-blood stimulations were performed for 7 patients at baseline, on-panobinostat (day 28), and at follow-up. Plasma measurements of IL-1β levels were done using a high-sensitivity Ella platform (ProteinSimple; R&D Systems). We used single cytokine cartridges for IL-1β according to protocol, using plasma (EDTA tubes) from baseline, on-panobinostat (day 4), and follow-up, and stored them throughout the study at −80°C.

### Gene expression microarrays.

The effect of panobinostat on gene expression was evaluated using an Affymetrix Human Transcriptome 2.0 array (42). Briefly, using PAXgene blood RNA tubes (PreAnalytiX), five million PBMCs were directly lysed following isolation. Samples were stored at −80°C. RNA isolation, RNA integrity verification, hybridization to the Affymetrix Human Transcriptome 2.0 array (70,523 probes), and realigning were performed (Aros Applied Biotechnology, Aarhus, Denmark). Gene expression was evaluated at baseline (visit 2), day 4 on-panobinostat (visit 4), and follow-up (visit 12) for all patients. Gene expression data were normalized using Robust Multi-Array Average (RMA) algorithms (43).

### Statistical analysis.

We analyzed flow cytometry and whole-blood cytokine data using either a paired *t* test or a Wilcoxon matched-pair signed-rank test comparing baseline data to data from specific on-panobinostat time points. Correlations were determined using Pearson or Spearman analyses. Two-tailed *P* values below 0.05 were considered significant. GraphPad Prism (GraphPad Software, Inc.), version 6, was used for statistical analysis and for graphical presentation of T cell and cytokine data. For gene expression analysis in microarrays, the *LIMMA* (44) method was used for pairwise detection of differentially expressed gene transcripts; gene expression changes with a fold change value of >1.5 and a false-discovery-rate (FDR)-adjusted *P* value of <0.05 were considered significant. Principal-component analysis (PCA) of microarray data was performed using the “prcomp” function in R (45), and the WARD method implemented in R was used for unsupervised hierarchical clustering analysis of gene expression data as visualized in the presented heat maps. Spearman correlations were used to identify correlations (*P* < 0.05) between fold changes of gene expression data (log_2_) and corresponding fold changes of unspliced HIV-1 RNA data. Functional annotations of microarrays data were determined by Ingenuity pathway analysis (IPA). Graphical presentation of gene expression data as heat maps and Circos plots was carried out using R.

### Accession number(s).

All data were deposited in a public repository (GEO) and can be accessed via accession no. GSE109792.
